# Recommendations for life-cycle assessment of recyclable plastics in a circular economy

**DOI:** 10.1039/d4sc01340a

**Published:** 2024-05-24

**Authors:** Sarah L. Nordahl, Corinne D. Scown

**Affiliations:** a Energy Technologies Area, Lawrence Berkeley National Laboratory 1 Cyclotron Road Berkeley CA 94720 USA cdscown@lbl.gov; b Joint BioEnergy Institute 5885 Hollis Street Emeryville CA 94608 USA; c Biosciences Area, Lawrence Berkeley National Laboratory 1 Cyclotron Road Berkeley CA 94720 USA; d Energy & Biosciences Institute, University of California Berkeley CA 94720 USA

## Abstract

Technologies that enable plastic circularity offer a path to reducing waste generation, improving environmental quality, and reducing reliance on fossil feedstocks. However, life-cycle assessment (LCA) methods commonly applied to these systems fall far short of capturing the full suite of advantages and tradeoffs. This perspective highlights inconsistencies in both the research questions and methodological choices across the growing body of LCA literature for plastics recycling. We assert that conducting LCAs on the basis of tonnes of waste managed *vs.* tonnes of recycled plastics yields results with fundamentally different conclusions; in most cases, analyses of recyclable plastics should focus on the unit of recycled product yielded. We also offer straightforward paths to better approach LCAs for recycling processes and plastics in a circular economy by rethinking study design (metrics, functional unit, system boundaries, counterfactual scenarios), upstream assumptions (waste feedstock variability, pre-processing requirements), and downstream assumptions (closed-loop *vs.* open-loop systems, material substitution). Specifically, we recommend expanding to metrics beyond greenhouse gases by including fossil carbon balances, net diversion of waste from landfill, and quantity of avoided plastic waste leakage to the environment. Furthermore, we highlight the role that plastic waste plays as a problematic contaminant in preventing greater diversion of all wastes to recycling, energy recovery, and composting, suggesting that plastics may hold a shared responsibility for the system-wide greenhouse gas emissions that occur when mixed wastes are landfilled.

## Abstract 2

Life-cycle assessment can better capture the impacts of plastics recycling by expanding beyond greenhouse gases to include fossil carbon balances, net diversion of waste from landfill, and avoided release of plastic pollution to the environment.

## Graphical abstract

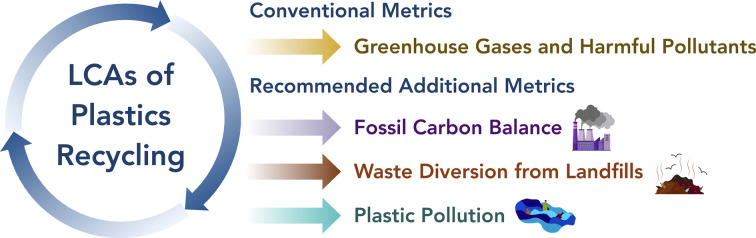

## I. Introduction

Plastics have become vital to the functioning of modern society, but they also present an enormous waste accumulation, resource depletion, and ecological challenge. A paradigm shift towards sustainable and circular management of plastics is necessary to mitigate these impacts. There has been a rapid expansion in the volume of research aimed at increasing the quantity and quality of recycled plastics.^1–8^ Recycling technologies and infrastructure for waste recovery (*i.e.* collection and sorting) are key to this transition,^9^ yet the methods by which different options can be evaluated and compared are nascent and inconsistently applied. The field of green chemistry has historically relied on rudimentary process-specific metrics such as the environmental factor and energy economy coefficient.^10^ Life cycle assessment (LCA) is a powerful and more holistic approach for evaluating the environmental footprint of production pathways and end-of-life management. However, conventional LCA approaches, which track material flows and quantify environmental impacts from cradle-to-grave, are most straightforward to apply to linear systems. There is less consensus in the research community on how to apply standard LCA methods to recycling and other more circular systems, resulting in inconsistent and misleading conclusions.

This article characterizes the weaknesses and challenges associated with quantifying and comparing the environmental impacts of circular plastic systems and provides recommendations to bridge the gap between green chemistry and LCA, while improving the robustness and adaptability of these methods. Recognizing the inherent challenges posed by circularity within plastic recycling systems, we present a methodological framework that effectively addresses study design (metrics, functional unit, system boundaries, counterfactual scenarios), upstream assumptions (waste feedstock variability, pre-processing requirements), and downstream assumptions (closed-loop *vs.* open-loop systems, material substitution). Past reviews have described many of these methodological choices, identifying uncertainties and demonstrating their significance on final results.^11–14^ This perspective summarizes and extends that prior work to offer a more comprehensive guide for approaching LCAs of circular plastic systems.

Our core critique is that there remains a lack of consensus regarding the most relevant research questions, metrics, and environmental impacts for circular plastics. While many LCAs focus primarily on greenhouse gases (GHGs) and global warming potential (GWP), other potentially significant impacts receive limited and inconsistent attention.^2,7,8,15,16^ Even studies that do include a wider range of midpoint impact categories (human toxicity potential, eutrophication potential, abiotic depletion potential, *etc.*) generally emphasize GWP as the most relevant environmental impact.^14,17–21^ The focus on GWP is likely the result of data limitations and broader government focus on decarbonization rather than the relevance of GWP in valuing different plastic recycling systems. Identifying a tractable collection of relevant metrics that are straightforward to quantify under limited data availability and better capture the main environmental value proposition would be very valuable to the circularity and plastics research field as a whole. In addition to providing guidance for specific methodological choices, this perspective offers an important reframing of the role of plastic recycling and circularity in sustainable development.

## II. Designing better studies

### A. Relevant environmental metrics

**Fig. 1:**
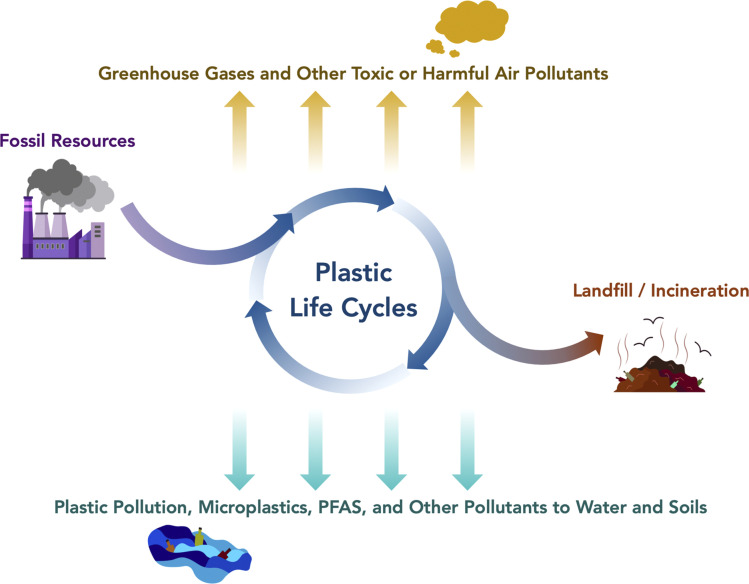
Environmental impacts from plastic life cycles. PFAS refer to per-and polyfluoroalkyl substances. Figure created in part with https://www.BioRender.com.

The role of plastic recycling in sustainable development needs reframing. While some processes do meaningfully reduce GHG emissions compared to fossil-based virgin plastic production, the magnitude of the impact is limited. For context, virgin plastic production contributes less than 2% to total annual GHG emissions from the US.^8,22^ Arguably, public funds spent to subsidize plastic circularity can achieve greater GHG mitigation if redirected toward the development of renewable energy. This begs the question: why devote resources to plastic circularity, and how can the value of such efforts be better quantified? GHG emissions and GWP are not the only environmental issue associated with plastic consumption that may be addressed with improved material circularity (Fig. 1). Beyond GHG emissions and other conventional midpoint indicators, we argue for the addition of three metrics in LCA to capture the goal of transitioning from linear to circular plastics: (1) net fossil carbon balance, (2) net waste diversion from landfills, and (3) net avoided or mitigated plastic waste released to the environment.

A commonly cited impact of a linear make-take-discard plastics system is the continued reliance on petrochemicals.^23^ Ethylene, for example, is an input to low-density polyethylene (LDPE/LLDPE), high-density polyethylene (HDPE), and polyethylene terephthalate (PET), and it is a product of naphtha and ethane crackers. As the global transition away from fossil fuels continues, the market and emissions impacts of continued reliance on these feedstocks is highly uncertain.^23^ Integrated assessment models, including the Global Change Analysis Model (GCAM) and the Integrated Model to Assess the Global Environment (IMAGE), have only recently attempted to capture the emissions and resource impacts of a drawdown in fuel demand paired with continued or increased demand for petrochemicals.^24,25^ In the near term, it is possible to draw from the burgeoning field of carbon accounting to create a simpler metric: net fossil carbon balance. Such a balance should include loss rates throughout the system, non-recycled solvents, and all other fossil inputs that are not recovered for productive use. While some studies report net fossil resource depletion,^2,7,26,27^ we recommend offering a more transparent breakdown of the fossil carbon balance. Ideally, such a metric should separate direct fossil carbon use from indirect to distinguish between an inherently fossil carbon-reliant process and one that requires electricity that is, for the time being, still partially reliant on fossil fuels. Even for bioplastics production, tracking fossil carbon inputs can be valuable because of the upstream fossil energy and fertilizer requirements.^28^ This approach is not without pitfalls; for example, the carbon contained in a low-value char produced from pyrolysis could arguably be treated as a waste or a product, thus impacting the overall fossil carbon balance. Transparently documenting such underlying assumptions can partially address these concerns.

Aside from reducing reliance on fossil feedstocks, another commonly cited concern is accumulation of plastic waste in landfills. Plastic recycling technologies can offer a viable alternative to landfilling. However, not all technologies achieve this equally effectively; a recycling process that requires clear PET bottles will draw from a stream that is already commonly recycled in many countries.^3,29,30^ Conversely, a recycling process capable of handling plastic films is drawing from a stream for which few other viable alternatives exist.^31,32^ The fraction of plastic waste landfilled also varies widely by country, and even region-to-region. For example, the US landfills over 70% of plastic waste while the EU on average landfills less than 25%.^9,33–35^ Much in the same way that LCAs must include regionally-specific data for electricity grid mixes, future analyses would be well served to identify the baseline recovery, recycling, landfilling, and incineration rates for their location(s) of choice by considering regional infrastructure and management practices.^36^ By adding net change in landfilled waste as metric relative to a defensible counterfactual, LCAs can begin to place appropriate value on the development of technologies that target materials that are truly destined for landfills. Quantifying this value treats accumulation of waste in landfills as a worthy environmental metric on its own, separate from resource use and emissions. This metric should also incorporate waste generated during the recycling process itself, if that waste is landfilled.

Up to this point, we have argued that resource circularity and waste accumulation are potentially more relevant than GHG emissions. However, there are other emissions to the environment that are potentially more relevant to plastics, specifically. For the most part, current LCA literature has not quantitatively addressed the release of plastics into the environment and the accumulation of microplastic pollution and per-and polyfluoroalkyl substances (PFAS). It is known that microplastics and associated PFAS are persistent pollutants that bioaccumulate and harm both ecosystem and human health,^37–39^ but the source-receptor and dose-response relationships remain highly uncertain.^40^ Despite a general consensus in the scientific community that plastic pollution is harmful, the full human health and ecosystem impacts of microplastics, as well as the distribution of these impacts among communities, are not yet well understood, much less captured in any defensible reduced-form model analogous to those used for air quality impacts.^41,42^

Simply attempting to identify processes that generate and release microplastics (particles that are smaller than 5 mm) for the system being studied (*e.g.* milling, extrusion, pressure washing, weathering, tire wear on roads) can be a meaningful first step.^7,40^ One recent study did explore the generation of microplastic pollution over plastic life-cycles and found that, while recycling scenarios have reduced pollution impacts due to increased recovery, mechanical recycling facilities themselves contribute to microplastic pollution.^7^ In addition to processes that directly release microplastics (primary sources), it is important to consider leakage of larger plastic pieces to the environment that may degrade into microplastics with environmental weathering (secondary sources).^40,43,44^ This is a field worthy of further study. Until better tools and methods exist for capturing the full impacts of plastic pollution, a simpler solution may be to estimate the net avoided plastic waste (including microplastics) released to the environment. This will also require the collection and synthesis of new data. Much like the net mass of material diverted from landfills, mass of avoided plastic pollution is highly location dependent and is subject to the greatest data quality/availability limitations. Some regions or countries may have a record of improper/illegal dumping and this higher likelihood of leakage to the environment should be captured. Conversely, systems that harvest ocean plastics or divert plastic waste streams with high leakage potential could be assigned the equivalent of plastic pollution offsets. However, by including even this simplistic plastic pollution metric, it is possible to gain insights into a system's contribution to environmental quality beyond climate change. Tracking these metrics will hopefully provide a motivation to collect, synthesize, and publish supporting datasets necessary to reduce their uncertainty. They also provide a foundation upon which more sophisticated plastic pollution metrics may be built as our collective understanding of microplastics pollution impacts evolves.

### B. Functional unit and system boundaries

**Fig. 2:**
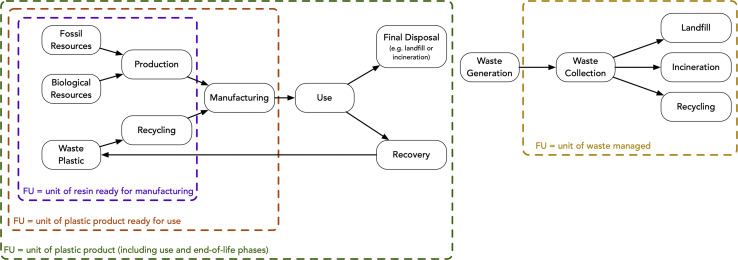
Example system boundaries for various functional units (FU: functional unit).

A clearly defined functional unit and system boundary are equally important as the selection of environmental metrics in any LCA; this is particularly challenging in systems that handle and convert wastes. For plastic recycling systems, there are typically two main types of functional units: production-based (unit of plastic produced) and waste intake-based (unit of waste managed) (Fig. 2). Studies using production-based functional units generally compare recycling strategies against virgin plastic production. Studies using waste management functional units focus on evaluating a recycling process against other waste management options (*i.e.* landfilling, incineration, and/or other recycling).

When the primary goal of a system is to achieve circularity and produce a high-quality recycled product, a production-based functional unit is likely to be more appropriate. When a system is built with the intention of deriving value primarily from the waste it takes in and treats, and the waste is converted to lower-value products (*e.g.*, steam, electricity, char, marine fuel), a waste intake-based functional unit can be appropriate. A simple check based on economics can elucidate which of these cases best describes a given system: are waste intake/tipping fees the primary source of revenue or is more revenue generated from the finished product(s)? Using a waste intake-based functional unit when the primary value of a process is in the quantity of waste it accepts has the added advantage of avoiding complex allocation approaches across a variety of products (some of which may have near-zero value). However, waste intake-based LCAs are most relevant for systems that do not achieve something close to circularity. For example, plastic pyrolysis processes are most easily compared based on a waste intake function unit, as otherwise comparable pyrolysis facilities may opt to use their outputs for different purposes (*e.g.*, fuels or petrochemical feedstocks) depending on local market conditions. When possible, we recommend opting for a production-based functional unit for systems that are oriented toward maximized recycling/circularity, particularly when the type(s) of output are likely to remain consistent.

Among production-based LCAs, there is variability in the system boundary definition and final cut-offs (Fig. 2 and Table 1). Weaknesses and strengths associated with the different options for functional units and associated system boundaries are listed in Table 1. Choosing an appropriate functional unit can be dependent on data availability. Using the widest system boundary (as depicted by the green box in Fig. 2) requires the most data and understanding of real world market behavior and infrastructure availability. In many cases, the data is simply not available and a less expansive system boundary may be justified.

**Table 1:** Types of functional units

Functional unit	Cut-off	Strengths	Weaknesses
Unit of resin ready for manufacturing	Before manufacturing	● Aligns with traditional LCA practices used for linear systems	● Does not address reusability or recoverability of plastic at end-of-life
		● Does not require any assumptions about use phase for plastic material, product form, or recoverability	● Neglects manufacturing impacts and the downstream stages of the plastic life cycle, including waste management and potential circularity options
Unit of plastic product ready for use	Before use-phase	● Aligns with traditional LCA practices used for linear systems	● Neglects the downstream stages of the plastic life cycle, including waste management and potential circularity options
		● Easy to compare different plastic products with the same function	
Unit of plastic product (including use and end-of-life phases)	Full system	● Accounts for full life-cycle including waste management and potential circularity options	● Difficult to know/represent real-world variability in operations and plastic recovery
		● Incorporates feedback loops	
Unit of waste managed	End-of-life only (gate-to-grave)	● Provides insights into the environmental impacts of different waste management scenarios (*e.g.* recycling, incineration, and landfilling)	● Comparing products with different waste streams can be challenging
			● May require more complex system boundaries and data due to diverse waste management practices

### C. Counterfactual scenarios

As is the case with any waste-based LCA, the fundamental question is: how would the material have been managed in a business-as-usual scenario? The counterfactual represents what would have occurred if the system in question did not exist. Appropriate counterfactuals are region-specific and should reflect available infrastructure and most likely management for the study area.^36^ It is important to note that “failure to recover” or “leakage to the environment” is almost always an inappropriate counterfactual, and may overestimate the net benefits of a given recycling system. Incineration and landfilling are the most common counterfactuals for plastic waste in most recycling studies.^13,14,45,46^ The incineration counterfactual is strongly dependent on what is displaced by the resulting energy generated. For example, in a recent LCA of plastic recycling, Jeswani *et al.* assumed that the resulting electricity from incineration with energy recovery offset the German grid mix in 2030 (mostly wind, solar and natural gas electricity).^47^ However, many countries are increasing the share of renewable electricity generation on their respective grids and this will decrease the value of incineration with energy recovery, assuming it offsets a mostly-clean grid mix.

**Fig. 3:**
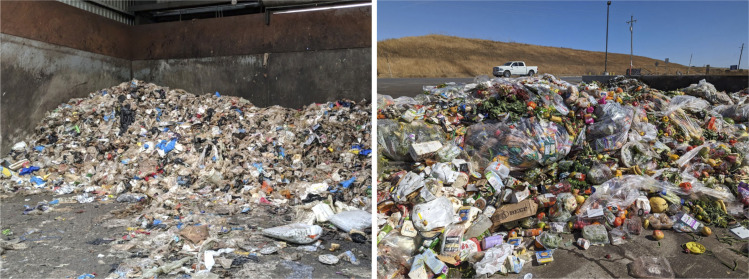
Images of plastic contamination in organic waste streams. Photos taken at Zero Waste Energy Development Company in San Jose, CA and Yolo County Central Landfill in Woodland, CA. Photo credit (both): Corinne Scown.

The landfilling counterfactual appears comparatively simple at face value; plastic does not rapidly degrade to methane in the manner that food waste and other organics do, so its direct contribution to fugitive methane emissions is negligible. One could argue that landfilling of plastic waste stores carbon and is preferable to incineration, but as a contributor to plastic pollution and other environmental issues, landfilling is not an efficient or sustainable means of storing substantial quantities of carbon.^27,48^ Furthermore, the presence of plastic waste in municipal solid waste streams adds cost and complexity to any organic waste recovery efforts (*e.g.*, composting or anaerobic digestion). Plastic contamination must be separated from organic waste using depackaging machines and other physical separation strategies, increasing the likelihood that mixed organic waste streams will be deemed too costly to process (Fig. 3). High plastic contamination rates result in rejection of mixed wastes from composting facilities.^49^

The standard practice in LCA is to calculate material-specific landfill emission factors using models such as the U.S. Environmental Protection Agency's Waste Reduction Model (WARM). ^50–52^ This places the full burden of landfill methane emissions on organic waste that degrades rapidly. For example, Nordahl *et al.* (2023) presents an LCA of polypropylene recycling where the basecase counterfactual is virgin polypropylene production with landfilling, but landfilling is assumed to have negligible GHG impacts. Here, we argue that such an approach may underestimate the role of plastic in perpetuating high rates of landfilling. Because plastic waste is commingled with organic waste, it indirectly contributes to landfill GHG emissions by affecting organic waste diversion rates. LCA researchers could consider additional counterfactual scenarios where landfill methane emissions are attributed to plastic waste based on the mass fraction of total landfilled waste. Future consequential LCAs could go several steps further to explore the causal relationship between plastic contamination rates and waste diversion from landfills.

A final consideration in selecting appropriate counterfactuals is the baseline recycling rate. Not all types of plastic are produced or managed at their end-of-life in the same way. For example, consider the study design for a production-based LCA of a new, advanced recycling system for PET bottles. PET bottles are already recycled at comparatively high rates. In the US, about 30% of PET bottles are mechanically recycled,^53^ so assuming a counterfactual of 100% landfilling for PET is likely not appropriate. For other types of plastics that are commonly not recycled (*e.g.* polypropylene is recycled at a rate of less than 1% in the US), landfilling may indeed be the appropriate counterfactual assumption.^4^

## III. Upstream assumptions

Implementing more circular plastics systems requires handling an increasingly variable set of feedstocks. Most recycling technologies are polymer-specific and require fairly pure input streams.^4^ In short, they are not tolerant to contamination from other plastics or non-plastic materials; some contaminants (such as metals or chlorine-containing compounds) may be more problematic than others. Understanding the nature of likely plastic waste feedstock streams is important because the level of contamination in an input waste stream to a recycling process and the associated need for preprocessing can have a substantial impact on the final LCA results of a circular plastic system.^13^

### A. Waste feedstock variability

Accurate modeling of real-world plastic sorting and recycling is difficult because of limited data availability and the inherent variability of plastic waste streams. Variations occur not only across different locations but also over time, making it challenging to establish a standard “typical” feedstock for analysis.^54^ In some places (*e.g.* many European countries), there is a high degree of source separation and plastic waste is sorted from non-plastic waste by consumers.^55^ In the US, there is far less source separation and recoverable plastic waste often ends up commingled with other recyclables in centralized sorting facilities.^55^ Even within a country, waste management services can vary municipality-to-municipality. Urban areas with high population density tend to have more waste management and recycling infrastructure in contrast to rural areas that have less infrastructure and may not recycle at all.^56^ In the case of centralized sorting, material recovery facilities (MRFs) take in mixed recyclable waste streams and separate plastic materials by polymer type. Currently, of plastic types, most US MRFs only target PET and HDPE with a particular focus on bottles and other rigid forms.^3,29^ Other types of polymers are baled together and can be routed for further sorting and processing, but are more commonly sent to landfills.^57^ Feedstock streams for recycling facilities are generally baled outputs from MRFs, or these could be produced by secondary sorting facilities that take in mixed bales. In an LCA of a plastic recycling system, it is important to identify appropriate assumptions for the incoming feedstock stream, including the type of MRF bale and average bale composition. We recommend using rigorous sensitivity analysis to capture the variability and parameter uncertainty associated with collection schemes (which includes waste transportation and MRF sorting) and the composition of available plastic waste bales. To capture prospective changes in waste generation, collection and baling, scenario analysis can capture potential future impacts.

### B. Plastic waste pre-processing

Although MRFs tend to use physical sorting processes that require modest quantities of electricity per unit of waste processed, outputs from MRFs are not clean enough to directly enter a recycling process because even low levels of contamination reduce product yields and output quality.^4,58^ Preprocessing is required, including shredding, washing, grinding, float-sink separation, and drying. These processes are considered to be a part of the mechanical recycling process which concludes with extrusion to produce recycled plastic. In the case of advanced recycling *via* solvent-based or chemical methods, preprocessing is likely to include all or most of those same processes, including extrusion, which can enable melt filtration and allows for continuous process flows.^4^ When incoming bales are very contaminated, these processes can contribute to higher emissions and lower output yields. Despite the importance and impact of preprocessing on LCA results, some past studies only consider aspects of preprocessing and exclude energy-intensive processes like extrusion, assuming idealized conditions or relatively pure feedstock streams that better reflect lab-scale testing than real world conditions.^59^ In future studies, it is essential that researchers are transparent about their assumptions and consider the full extent of pre-processing required to convert typical incoming waste streams to clean streams ready for recycling.

## IV. Downstream assumptions

Producing interpretable LCA results requires a clearly defined function unit and, to this point, we have emphasized the value of production-based functional units. However, recyclate (the output from recycling processes) varies in quality depending on the type of polymer being recycled, composition of the input waste mix, and type of recycling technology.

### A. Closed-loop *vs.* open-loop feedback and allocation

In circular systems, materials and resources can be cycled back into the system, creating complex feedback loops that are not always easily accounted for in an LCA model. Closed-loop recycling systems are based on material continuity, transforming post-consumer plastics directly back into the same product (or product category) with minimal quality loss. Open-loop recycling, in contrast, represents a broader approach.^60^ Some plastic waste may be “downcycled” into lower-value plastic applications.^61,62^ In other cases, plastic waste may be pyrolyzed to produce monomers as inputs for petrochemical processing.^63^ This flexibility creates an open loop, where materials exit their original product category but retain at least some of their valuable utility.

Closed-loop feedback is simpler to model using a conventional LCA approach. If the quality of recyclate is close to that of virgin material, defining an appropriate functional unit is straightforward and the entire system with and without recycling can be directly compared.^64^ If the system boundaries include closed-loop feedback of a particular product, then steady state can be assumed to assess total input (equal to initial input minus recycled output) or surplus output (equal to recycled output minus initial input).

Unlike the closed-loop ideal, where recycled materials directly replace virgin inputs, open-loop systems involve diverse recycling pathways with varying outputs. Open-loop recycling systems are a well-known allocation challenge in LCA.^64^ Establishing a common output-based functional unit that makes recyclates directly comparable to virgin resin is challenging and may involve arbitrary decisions to be made regarding how much of the virgin material's burden should be allocated to the outputs of recycling (*e.g.*, the 50/50 method, cut-off method).^11,28,65,66^ Other allocation methods for open-loop recycling (*e.g.*, substitution method, loss of quality method, circular footprint formula) attempt to capture what is a known issue for plastics: varying quality of recycled material.^11,28,65–70^ Mechanical recycling can yield lower-quality downcycled materials or material that is not approved for specific applications (*e.g.*, food contact materials), whereas advanced chemical or solvent-based methods may produce higher quality recyclate. Accurately capturing the spectrum of quality in recyclates adds complexity because the quality requirements for plastics are so application specific and not as standardized as some other materials, such as steel.^71^ To address this problem for plastics, some LCA practitioners have employed material substitution factors.^4,7,12,13,66,72^ However, even attributing environmental benefits solely based on material substitution factors can be misleading, as the substitution factor and resulting life-cycle impact vary based on the intended application and are uncertain due to dynamic market conditions.^13^

### B. Material substitution

**Fig. 4:**
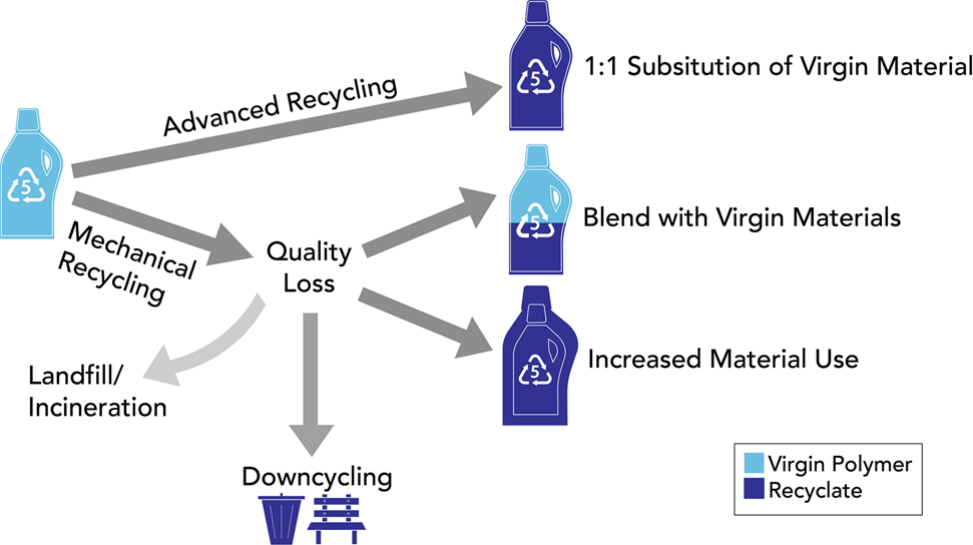
Substituting virgin polymer with recyclate.

While it is possible that a recyclate may displace materials other than its virgin counterpart, the standard research approach has been to assume that recycled plastics will only offset other recyclates or their virgin counterpart.^73^ In some cases, simply assigning credits to a recycled material to its virgin counterpart on a 1 : 1 basis risks overestimating its environmental benefit. Recycled plastics can exhibit inferior physicochemical properties compared to virgin resins, necessitating blending with virgin material to achieve desired material performance goals (Fig. 4).^4,19,74^ Blending limits vary based on type of recyclate (accounting for polymer type and recycling process) and application. In most cases, we do not recommend using any substitution factor based on blending limits because there is likely surplus market capacity for absorbing and blending recycled plastics.^75^ Until the industry-wide capacity has been reached, recycled plastics could displace their fossil counterparts on a 1 : 1 basis for specific blended applications. However, inferior quality in recycled plastics can also mean more material is required to make a particular product from recyclate relative to using virgin resin (Fig. 4).^4,15^ In this case, a 1 : 1 displacement assumption would be inappropriate.

This figure depicts how quality loss during plastic recycling can affect the substitution of virgin polymer with recyclates. Note that only quality loss (as opposed to mass loss or yield) from recycling is shown. This figure is adapted from Nordahl *et al.* (2023).^4^

Adding further complexity to this issue is the possibility of rebound effects in the market. It is possible that the production of recycled plastics does not substantially impact the use of fossil-based plastics, but instead, only contributes to the growing use of plastic products.^76^ A consumer, for example, may opt to purchase a product advertised as being made from recycled plastic instead of purchasing a non-plastic alternative (*e.g.*, paper, natural fibers, or wood). Because of the high uncertainty and product-to-product variation in appropriate substitution factors, we advise against using a single substitution factor for the purposes of comparing recycled plastic to the virgin alternative. If a study is focused on a specific application for the recyclate, a well justified substitution factor (or several factors) may be warranted. Justifications should consider factors like quality loss during recycling, the functionality of the recyclate, its intended use sector, and consideration of potential rebound effects.^12,73,77^

## V. Summary of recommendations and future outlook

The widespread application of LCA to evaluate and compare circular plastic systems is encouraging; systems analysis can offer important insights into which recycling strategies can yield the greatest societal benefits. At this juncture, researchers would be well served to think critically about what the goals of circular plastics systems are and how LCAs can better capture progress toward those goals. First, we recommend that researchers consider the role that plastics play in the broader waste management system as a contaminant that hinders diversion of other valuable streams; assigning plastics a share of total GHG emissions from landfilling mixed wastes is a practice worthy of consideration. We also urge a shift beyond the common focus on GHGs towards a more holistic perspective, encompassing other potentially significant but often neglected impacts. Metrics to address landfill diversion, net carbon recovery, and impacts on net plastic pollution can produce a far richer set of results. Air pollution and resulting human health impacts may be challenging to incorporate given lack of data and variations in the use of emissions control technologies, but these also offer an opportunity to capture non-GHG impacts; while impacts on local air quality may be minor compared to other sustainability benefits, there is evidence that increasing recycling rates may reduce municipal particulate matter emissions.^78,79^ To move beyond a narrow focus on material recovery and GHGs, we must acknowledge the intricate relationships between circularity and the wider environmental, economic and social landscapes. Recycling and waste infrastructure is highly regionally specific and it is important to recognize that impacts from plastic waste generation are not necessarily equitably distributed between communities or more broadly, between countries. This is one of the reasons that we advocate for aggressive transparency of assumptions, comprehensive scenario analysis, sensitivity analysis, and the inclusion of a plastic pollution metric. Future research in this area has an opportunity to help build a more nuanced understanding of the potential trade-offs involved and highlight the importance of integrating social justice and equity considerations into the analysis of circular solutions.

The recommendations outlined in this work are intended to pave the way for more robust and insightful LCAs. Embracing scenario analysis and sensitivity analysis is crucial. Exploring a wide range of scenarios encompassing current and potential future variations in system designs and counterfactuals fosters a comprehensive understanding of the environmental benefits of recycling. While applying generous diversion/offset/material substitution credits may seem appealing and easy to implement, expanding the scenario list provides a more nuanced picture. Supplementing LCA with rigorous sensitivity modeling can address the inherent uncertainty and/or variability in data and assumptions, leading to more realistic and defensible results. Lastly, transparency and meticulous documentation are paramount. Clearly articulating the chosen counterfactual scenario, data sources, and allocation methods ensures an LCA's reproducibility and interpretability. By employing the guidance presented here, LCAs can navigate the complexities of circular plastics, paving the way for a more accurate and insightful evaluation of recycling's true sustainability and environmental benefits.

## Author contributions

S. L. N. and C. D. S. conceptualized the article. S. L. N. conducted the literature review. S. L. N. and C. D. S. wrote the article.

## Conflicts of interest

C. D. S. has a financial interest in Cyklos Materials.
